# Downstream events initiated by expression of FSHD-associated DUX4: Studies of nucleocytoplasmic transport, γH2AX accumulation, and Bax/Bak-dependence

**DOI:** 10.1242/bio.059145

**Published:** 2022-02-22

**Authors:** Isabel F. Masteika, Anvitha Sathya, Sachiko Homma, Bess M. Miller, Frederick M. Boyce, Jeffrey Boone Miller

**Affiliations:** 1 Department of Neurology, Boston University School of Medicine, Boston, Massachusetts 02118, USA; 2 Biological & Biomedical Sciences Program, Harvard Medical School, Boston, Massachusetts 02115, USA; 3 Department of Neurology, Massachusetts General Hospital, Boston, Massachusetts 02114, USA

**Keywords:** BAX, DUX4, Facioscapulohumeral muscular dystrophy, γH2AX, Myotube, Phospho(Ser139)-H2AX

## Abstract

Abnormal expression in skeletal muscle of the double homeobox transcription factor DUX4 underlies pathogenesis in facioscapulohumeral muscular dystrophy (FSHD). Though multiple changes are known to be initiated by aberrant DUX4 expression, the downstream events initiated by DUX4 remain incompletely understood. In this study, we examined plausible downstream events initiated by DUX4. First, we found that nucleocytoplasmic protein export appeared to be decreased upon DUX4 expression as indicated by nuclear accumulation of a shuttle-GFP reporter. Second, building on studies from other labs, we showed that phospho(Ser139)-H2AX (γH2AX), an indicator of double-strand DNA breaks, accumulated both in human FSHD1 myotube nuclei upon endogenous DUX4 expression and in *Bax-/-;Bak-/-* (double knockout), SV40-immortalized mouse embryonic fibroblasts upon exogenous DUX4 expression. In contrast, DUX4-induced caspase 3/7 activation was prevented in *Bax-/-;Bak-/-* double knockout SV40-MEFs, but not by single knockouts of *Bax*, *Bak*, or *Bid*. Thus, aberrant DUX4 expression appeared to alter nucleocytoplasmic protein transport and generate double-strand DNA breaks in FSHD1 myotube nuclei, and the Bax/Bak pathway is required for DUX4-induced caspase activation but not γH2AX accumulation. These results add to our knowledge of downstream events induced by aberrant DUX4 expression and suggest possibilities for further mechanistic investigation.

## Abstract (precis)

**Summary:** Aberrant DUX4 expression initiates pathogenesis in facioscapulohumeral muscular dystrophy. These studies delineate additional downstream events that accompany aberrant DUX4 expression and suggest possibilities for further mechanistic investigation of FSHD pathogenesis.

## INTRODUCTION

Pathogenesis in facioscapulohumeral muscular dystrophy (FSHD) is due to aberrant expression, particularly in skeletal muscle, of the 424 amino acid, full-length isoform of the double homeobox protein DUX4. In the two forms of the disease – FSHD1 (∼95% of cases) and FSHD2 (∼5%) – the DUX4 transcript is produced from an open reading frame in the most telomeric 3.3 kb D4Z4 repeat at chromosome position 4q35 (Lemmers et al., 2010; 2012), with rare cases having aberrant transcription from chromosome 10 (Lemmers et al., 2022). In FSHD1, de-repression of *DUX4* expression is associated with fewer D4Z4 repeats, loss of epigenetic silencing, and a telomeric polyadenylation signal (Campbell et al., 2018; Gatica and Rosa, 2016; Haynes et al., 2018; Himeda and Jones, 2019; Wang and Tawil, 2021). In FSHD2, *DUX4* expression is also de-repressed, in this case due to mutation of *SMCHD1*, which leads to DNA hypomethylation (Lemmers et al., 2012).

DUX4 is a double homeobox transcription factor, and ectopic expression of full-length DUX4 (DUX4-FL) induces multiple molecular and cellular changes that may be linked to FSHD pathology through pathways that remain incompletely defined (Bosnakovski et al., 2017a; 2017b; Jones et al., 2020; Kowaljow et al., 2007; Mitsuhashi et al., 2013; 2018; Rickard et al., 2015). In addition to altering gene expression via its action as a transcription factor, DUX4-FL expression is associated with altered splicing of multiple mRNAs (Banerji et al., 2017; Jagannathan et al., 2016; Rickard et al., 2015). Furthermore, DUX4-FL expression leads to accumulation of dsRNA and nuclear aggregation of EIF4A3 (Shadle et al., 2017) and inhibits nonsense-mediated decay (Feng et al., 2015). DUX4 expression in FSHD1 myotubes also induces nuclear aggregation of proteins that regulate RNA homeostasis including TDP-43, FUS, and SC35 and disrupts ubiquitination and proteostasis (Homma et al., 2015; 2016; Mitsuhashi et al., 2018).

DUX4-FL-induced cytotoxicity requires intact homeodomains and a transactivating domain in the C-terminal region of the protein (Bosnakovski et al., 2017b; Choi et al., 2016; Corona et al., 2013; Geng et al., 2012; Mitsuhashi et al., 2013; 2018). A short DUX4 isoform (DUX4-S) consisting of just the N-terminal 159 amino acids (including both homeodomains) of DUX4-FL is not toxic (Geng et al., 2011; Homma et al., 2015). In myotubes formed from FSHD patient-derived myoblasts or iPS cells, DUX4-FL expressed from its endogenous promoter is detectable by immunocytochemistry in only a small percentage of nuclei (Haynes et al., 2018; Himeda et al., 2014; Homma et al., 2015; Jones et al., 2012; Snider et al., 2010).

In this study, we sought to further delineate downstream events initiated by DUX4. In particular, we (i) examined the relationship between DUX4-FL expression and nucleocytoplasmic transport, (ii) confirmed and extended previous studies (DeSimone et al., 2019; Dmitriev et al., 2016; Shadle et al., 2019) of DUX4-initiiated accumulation of the phospho(Ser139)-H2AX (γH2AX) histone, a marker for double-strand DNA breaks, and (iii) examined the role of Bax/Bak pathway in DUX4-initiated caspase activation and γH2AX accumulation. The results delineate additional downstream events that accompany aberrant DUX4 expression and suggest possibilities for further mechanistic investigation of FSHD pathogenesis.

## RESULTS

### DUX4 and nucleocytoplasmic transport

We first asked if DUX4-FL expression might disrupt nucleocytoplasmic protein transport in myogenic cells. In a previous RNA-seq study, DUX4-FL expression was found to affect mRNAs encoding proteins in the RNA nuclear transport pathway (Rickard et al., 2015). In addition, we found that DUX4-FL expression induces nuclear aggregation or clearance of TDP-43 (Homma et al., 2015; 2016), and others have shown that TPD-43 pathology disrupts nucleocytoplasmic transport in neural cells (Chou et al., 2018). To directly examine how nucleocytoplasmic protein transport might be affected by DUX4 expression, we used a ‘shuttle-GFP’ reporter in which GFP has been modified to contain both nuclear localization and nuclear export signals (Woerner et al., 2016). We expressed the shuttle-GFP reporter in primary human myogenic cells and determined if DUX4 expression altered the nuclear-to-cytoplasmic ratio of GFP fluorescence. This ratio can indicate if a treatment alters nucleocytoplasmic transport, e.g. the ratio will increase if a treatment disrupts nuclear export thereby leading to nuclear accumulation of the shuttle-GFP.

**Fig. 1.**
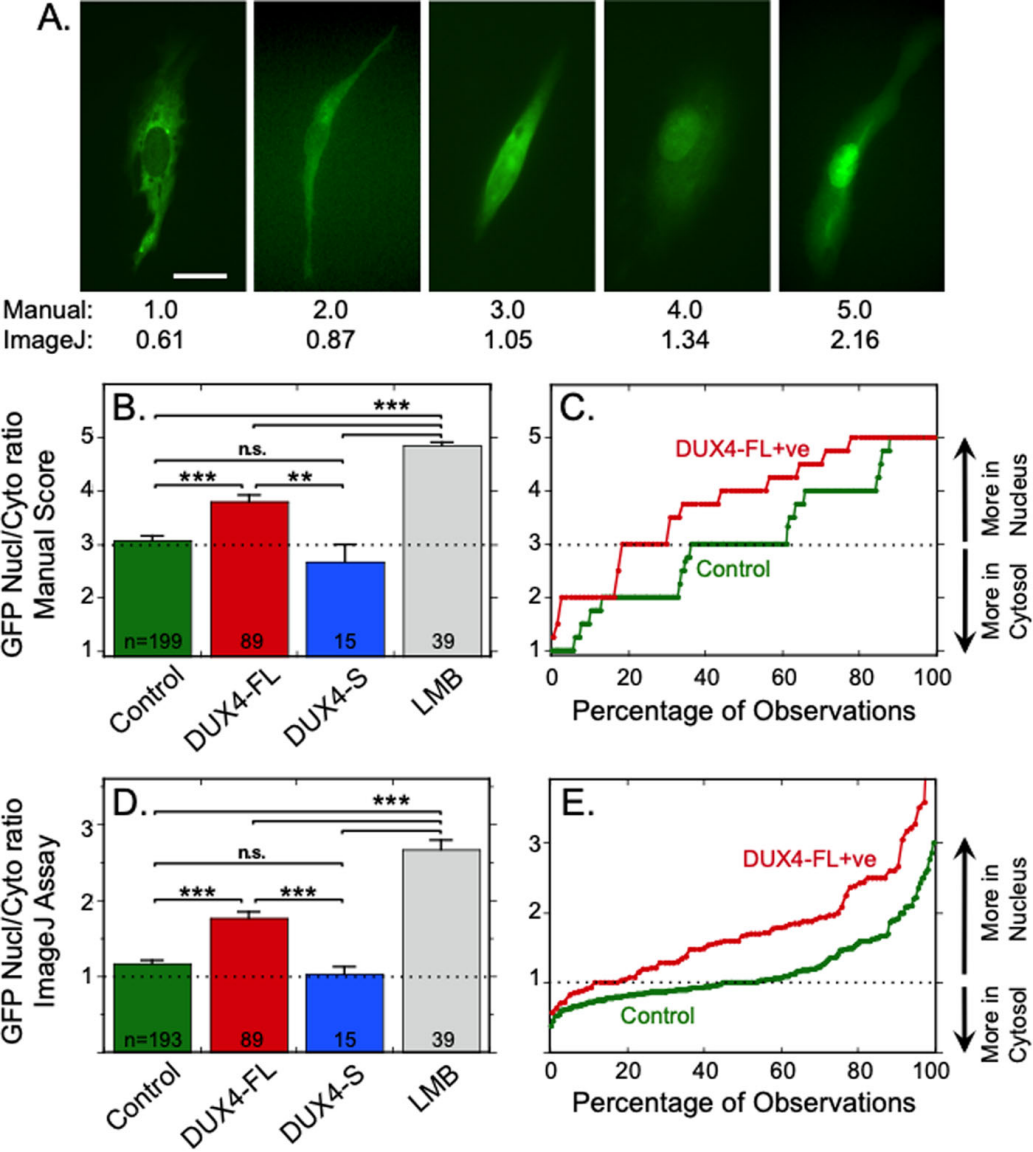
**Expression of DUX4-FL increased the percentage of myogenic cells with nuclear accumulation of a reporter for nucleocytoplasmic transport (shuttle-GFP).** (A) Examples of different patterns of fluorescence distribution in human 09Ubic myoblasts transfected with the shuttle-GFP reporter. The ‘Manual’ score below each image is the average score on a five-point scale (*n*=4 blind observers); and the ‘ImageJ’ score is the nuclear-to-cytoplasmic ratio of GFP fluorescence intensity. See Materials and Methods for descriptions of the Manual and ImageJ assays. Cells with scores of 4 and 5 were DUX4-FL-positive, see Fig. S1 for images of shuttle-GFP and DUX4-FL co-staining. Scale bar: 15 µm. (B) The percentage of myoblasts with average observer scores of ≥4 (i.e. in which fluorescence was more intense in the nucleus than the cytoplasm) was higher in cells that expressed DUX4-FL than in control cells with no DUX4 or in cells that expressed non-toxic DUX4-S. As a positive control, cells treated with Leptomycin B (LMB, 10 ng/ml for 3 h), an inhibitor of nuclear export, were almost uniformly given a score of 5, which was consistent with the expected accumulation of shuttle-GFP in nuclei due to failure of export. Cultures were examined at 48 h after addition of BacMam vectors (72 h after shuttle-GFP transfection). (C) Cumulative probability plot of manual scores showed that DUX4-FL-expressing myoblasts (green line) were more likely than control cells (red line) to have a higher score indicative of a higher nucleus-to-cytoplasmic GFP fluorescence ratio. (D) As in panel B, except showing the nuclear-to-cytoplasmic ratio of shuttle-GFP fluorescence obtained using the ImageJ program. (E) As in panel C, except showing the cumulative probability of the nuclear-to-cytoplasmic ratio of shuttle-GFP fluorescence measured with the ImageJ program. Panels B and D show mean±se, *n* as indicated; **P<0.01, ***P<0.001, n.s.=*P*>0.5 by ANOVA with Bonferroni post-tests. Dotted lines in panels B through E indicate scores where GFP fluorescence is approximately equal in nucleus and cytoplasm.

Indeed, we found that the percentage of cells with nuclear accumulation of the shuttle-GFP reporter was higher for DUX4-FL-expressing primary human myoblasts than in control cells that either lacked DUX4 expression or expressed DUX4-S (Fig. 1). For these experiments, cultures of control, unaffected primary human 09Ubic myoblasts were examined 48 h after addition of BacMam vectors (72 h after transfection of the shuttle-GFP expression plasmid). We obtained similar results using two different methods to assay shuttle-GFP distribution.

In the first method, blind observers (*n*=4) rated GFP fluorescence distribution in each cell on a five-point scale ranging from 1 (mostly cytoplasmic) to 5 (mostly nuclear) (Fig. 1A–C). The average observer score for cells that expressed DUX4-FL was higher than the average scores for cells with either no expression (at *P*<0.001) or expression of non-toxic DUX4-S (at *P*<0.01) (Fig. 1B). A cumulative probability plot showed that, though individual DUX4-FL-expressing cells were found with a range of average scores, the DUX4-FL-positive cells were overall more likely than control cells to have been assigned scores of 4 or 5 indicating increased nuclear accumulation of the reporter (Fig. 1C). Examples of shuttle-GFP distributions in DUX4-FL-positive myoblasts are given in supplemental data (Fig. S1). As a positive control, cells treated with Leptomycin B (LMB), a known inhibitor of nuclear export (Woerner et al., 2016; Wolff et al., 1997), had the highest average score, consistent with robust nuclear accumulation of the shuttle-GFP reporter (Fig. 1B). With this manual method, scores ≥4 were given to 100% of Leptomycin B-treated, 57% of DUX4-FL-expressing, 33% of DUX4-S-expressing, and 34% of control cells.

In the second method, we measured the average nuclear-to-cytoplasmic ratio of GFP fluorescence intensity within each cell using the ImageJ program. The results with the ImageJ method were similar to the results from the observer rating method. Primary 09Ubic myoblasts that expressed DUX4-FL showed a higher nuclear-to-cytoplasmic ratio of GFP fluorescence (equivalent to a higher observer score) than did myoblasts without DUX4 expression or that expressed DUX4-S (Fig. 1D). The cumulative probability plot again showed a range of values for DUX4-FL-positive cells, but the measured values were more often higher for DUX4-FL-positive than for control cells (Fig. 1E). Cells treated with Leptomycin B had the highest average nucleus-to-cytoplasm fluorescence intensity ratio, again consistent with inhibition of nuclear export leading to accumulation of the shuttle-GFP reporter (Fig. 1D). With the ImageJ method, a nuclear-to-cytoplasmic ratio of GFP fluorescence >1.2 was found in 100% of Leptomycin B-treated, 74% of DUX4-FL-expressing, 33% of DUX4-S-expressing, and 32% of control cells.

### DUX4 and γH2AX

We next confirmed and extended earlier studies (DeSimone et al., 2019; Dmitriev et al., 2016; Shadle et al., 2019) by examining how DUX4-FL expression affected localization of phospho(Ser139)-H2AX (γH2AX), a histone that is reported to accumulate at repairable double-strand DNA breaks (indicated by punctate immunofluorescence staining) as well as in nuclei of cells undergoing cell death (indicated by intense, more uniform nuclear immunofluorescence) (D'Abrantes et al., 2018; Georgoulis et al., 2017; Moeglin et al., 2019; 2021). Earlier studies from other groups with primary and immortalized myoblasts had found that exogenous DUX4 expression (plasmid-mediated or via doxycycline-inducible transgene) was accompanied by increased nuclear accumulation of γH2AX, as well as other markers of DNA damage (DeSimone et al., 2019; Dmitriev et al., 2016; Shadle et al., 2019). Additional evidence for DUX4-initiated DNA damage came from work showing increased numbers of TUNEL-positive nuclei (a marker for DNA fragmentation) in FSHD patient muscles and upon DUX4-FL expression in model systems (Hauerslev et al., 2013; Sandri et al., 2001; Statland et al., 2015; Wallace et al., 2011; Wuebbles et al., 2010).

**Fig. 2.**
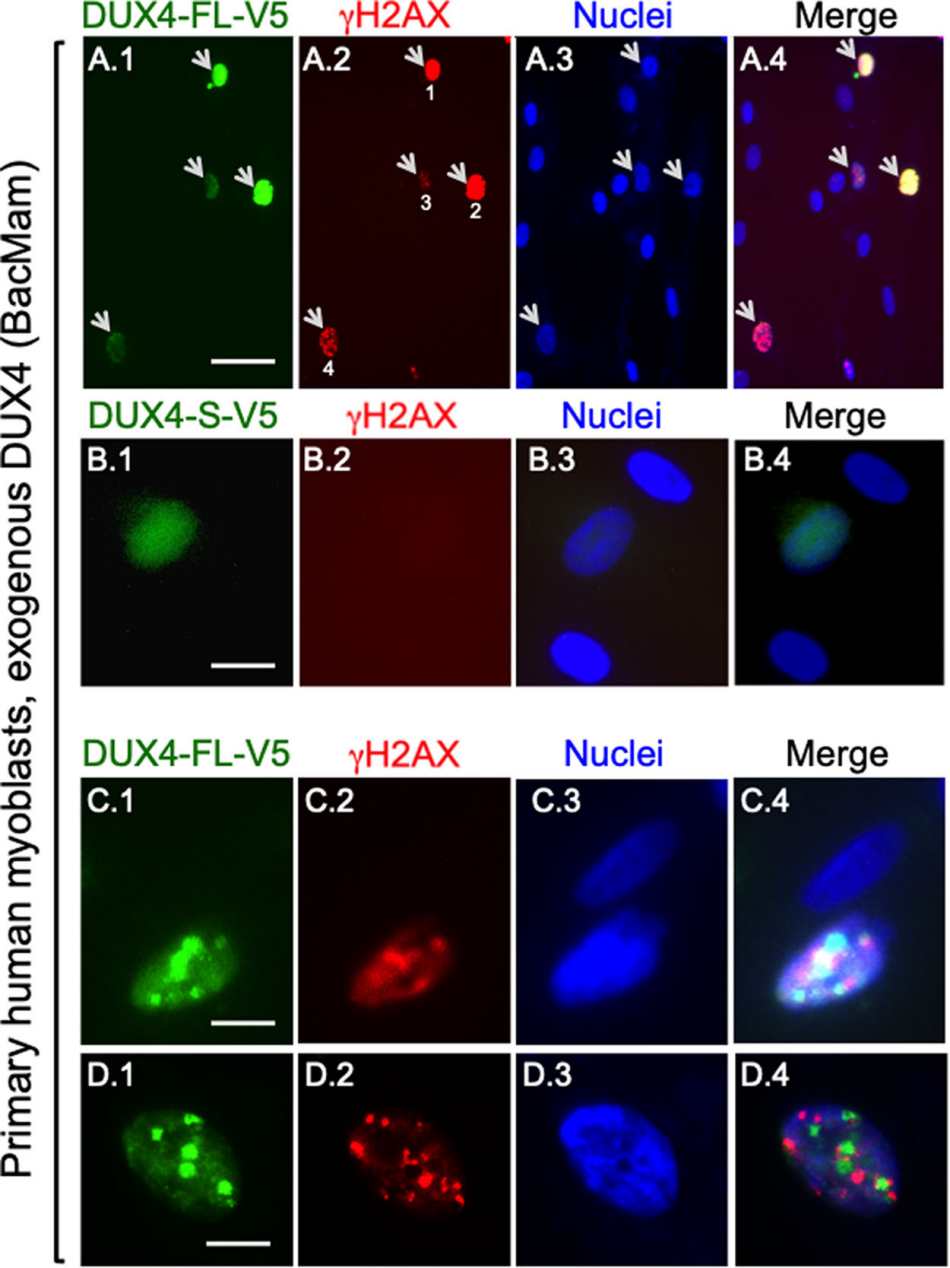
**In human myoblasts, exogenous DUX4-FL expression was accompanied by increased γH2AX immunostaining.** Cultures of control, unaffected primary human myoblasts (09Ubic) were incubated with a BacMam-DUX4-FL-V5 (rows A, C, D) or non-toxic BacMam-DUX4-S-V5 (row B) virus vector for 48 h then double-immunostained for the V5 epitope (i.e. DUX4) and γH2AX as indicated. A.1–A.4. Arrows indicate four myoblast nuclei that showed immunostaining for both DUX4-FL (green, mouse anti-V5 mAb) and γH2AX (red, rabbit mAb 20E3, PBS buffers). In contrast, the ten DUX4-FL-negative nuclei in this image did not show immunostaining for γH2AX. Scale bar in panel A.1: 40 µm. (B.1–B.4) In contrast to the increased γH2AX immunostaining seen upon expression of DUX4-FL, expression of the non-toxic, short isoform of DUX4-S was not accompanied by immunostaining for γH2AX in 09Ubic myoblasts. Scale bar in panel B.1: 17 µm. (C.1–C.4.) Higher magnification view of two 09Ubic myoblast nuclei, one of which showed punctate immunostaining for both DUX4-FL (red, rabbit-anti-DUX4-FL mAb E5-5) and γH2AX (green, mouse mAb 3F2). Scale bar in panel C.1: 12 µm. (D.1–D.4.) Additional example of an 09Ubic myoblast nucleus that showed punctate immunostaining for both DUX4-FL (green, mouse anti-V5 mAb) and γH2AX (red, rabbit mAb 20E3). Scale bar in panel D.1: 10 µm. In nuclei showing punctate immunofluorescence for both DUX4-FL and γH2AX, as in the two examples shown in rows C and D, there was little or no colocalization of the punctate DUX4-FL and γH2AX signals.

To confirm previous observations and to determine if a different method of exogenous expression produced similar results, we first examined γH2AX immunostaining in cultures of control, unaffected primary human myoblasts using BacMam-DUX4-FL for exogenous expression. Nuclear accumulation of γH2AX was more prevalent among primary human myoblasts that expressed DUX4-FL at 48 h after BacMam addition than in control cells that lacked DUX4 expression or expressed DUX4-S (Fig. 2A,B). In one study, for example, when all 09Ubic myoblasts in 12 40X fields were assayed, we found γH2AX immunostaining in 19 of 21 (90.5%) of the DUX4-positive nuclei, whereas of the 77 DUX4-negative nuclei only seven (9.1%) showed γH2AX immunostaining (and four of these had very weak staining). The DUX4-FL-positive and -negative groups differed at *P*<0.0001 (Fisher's exact test).

We then carried out additional double immunostaining studies to characterize in greater depth the relationship between DUX4 expression and γH2AX. As in previous studies in non-muscle cells (e.g. Moeglin et al., 2019), we found two patterns of γH2AX immunostaining in DUX4-FL-expressing nuclei. A subset of cells, typically <20% of the γH2AX-positive cells, had intense staining throughout the nucleus as exemplified by cells #1 and #2 in Fig. 2A.2. This pattern may indicate that the cell is irreversibly committed to cell death (Moeglin et al., 2019). More common was for the γH2AX immunostaining to appear as multiple foci or puncta, e.g., as in cells #3 and #4 in Fig. 2A.2 and the cells in Fig. 2C.2, D.2. This pattern may indicate that reversible DNA repair is underway (Moeglin et al., 2019; 2021). BacMam-mediated expression of DUX4-S, in contrast to DUX4-FL, was not associated with increased γH2AX immunostaining (Fig. 2B). As in previous studies (Homma et al., 2015; 2016), DUX4-FL immunostaining, though usually uniform, was punctate in a subset of nuclei. For those nuclei in which both DUX4-FL and γH2AX showed punctate staining, the DUX4-FL and γH2AX puncta appeared to be discrete with little or no colocalization (Fig. 2C,D), a result not previously reported.

**Fig. 3.**
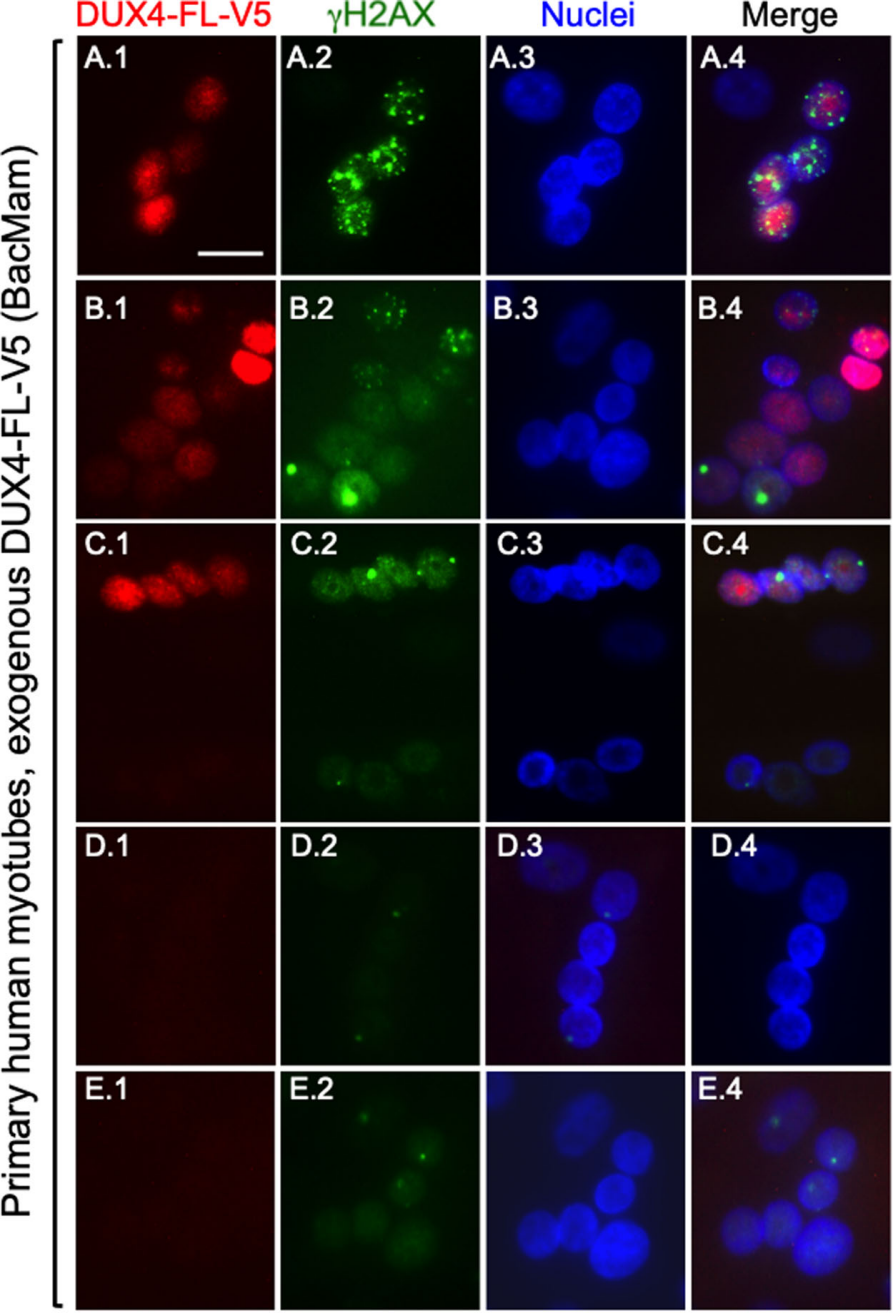
**Immunostaining for γH2AX was found more frequently in DUX4-FL-positive myotube nuclei.** Control unaffected, human primary myogenic cells were cultured for 7 days in differentiation medium at which time BacMam-DUX4-FL-V5 was added for an additional 2 days. Cultures were double immunostained for DUX4-FL (red, rabbit anti-V5) and γH2AX (green, mAb 3F2) as indicated. Multinucleate myotubes with nuclei that were positive for both DUX4-FL and γH2AX are shown in panels A and B, as well as in the upper half of panel C. Such double-positive nuclei were found in a large majority of the DUX4-FL-positive myotubes (see text for quantitation). In contrast, the nuclei in most DUX4-negative myotubes, as shown in the lower half of panel C, as well as in panels D and E, did not exhibit obvious intense or multiply punctate staining for γH2AX, though a weak overall stain and rare single puncta were observed in some cells. Scale bar in panel A1: 15 µM.

**Fig. 4.**
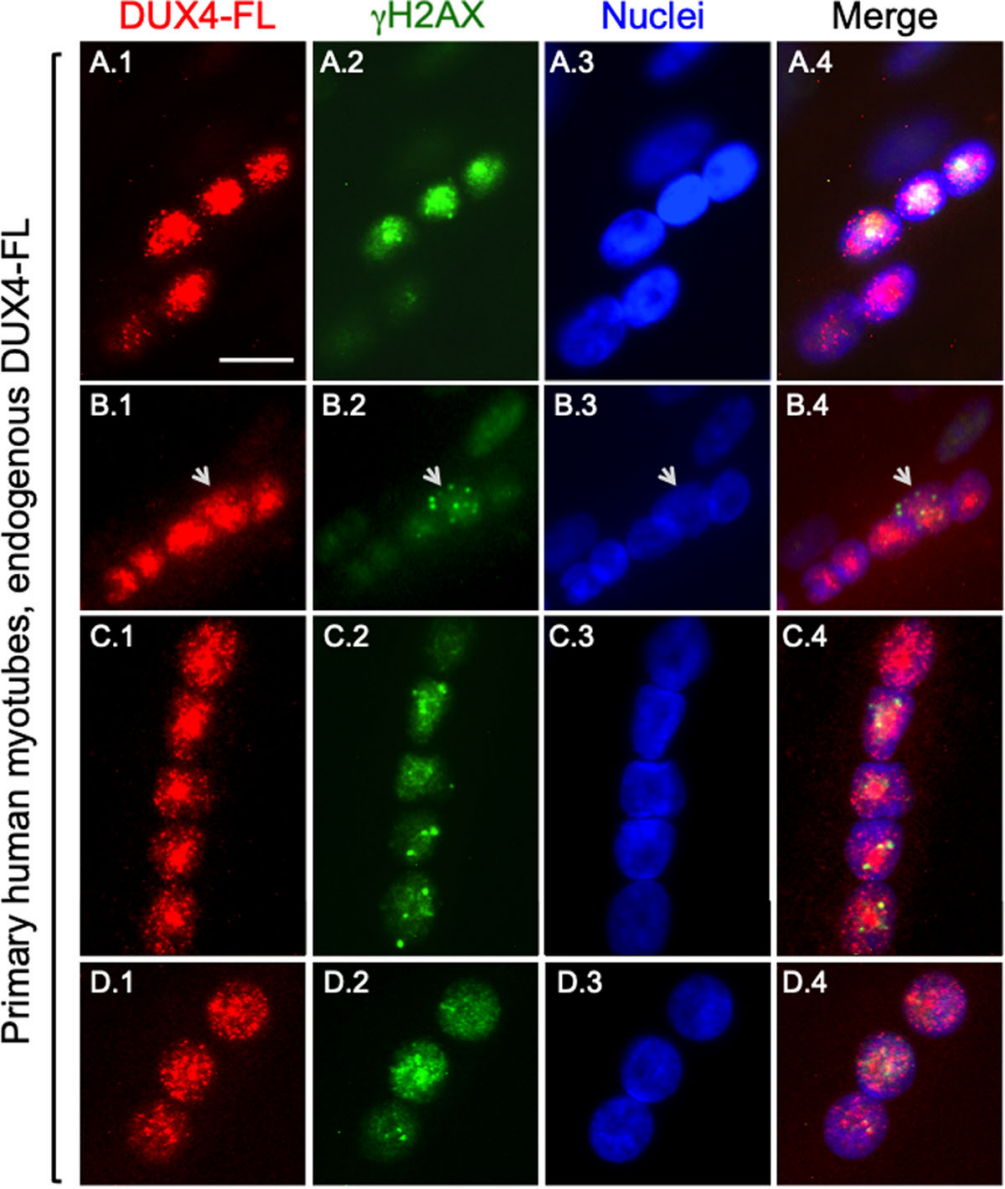
**Expression of endogenous DUX4-FL in myotubes formed from FSHD1 myoblasts was frequently accompanied by γH2AX immunostaining.** Human primary myogenic cells, termed 17Abic, obtained from a patient with FSHD1 were cultured for 7 days in differentiation medium to allow myotube formation and expression of DUX4-FL from the endogenous promoter. Cultures were double immunostained for DUX4-FL (red, mAb E5-5) and γH2AX (green, mAb 3F2) as indicated. In most multinucleate myotubes, all or almost all of the nuclei that expressed endogenous DUX4-FL also showed intense or multiply punctate immunostaining for γH2AX (see text for quantitation) (e.g. rows A, C, D). In some myotubes, however, the DUX4-FL-positive nuclei either did not show a γH2AX signal that was different from background (e.g. as in the lower two nuclei in row A), or the γH2AX foci were found in only a subset of the endogenous DUX4-FL-positive nuclei within a myotube (e.g. the one nucleus identified by an arrow in B). In contrast, as described in the text, a large majority of nuclei in most DUX4-negative myotubes did not show obvious staining for γH2AX (see text for quantitation), though a weak overall stain and rare single puncta were observed. Scale bar in panel A1: 15 µM.

Because previous immunostaining studies had focused only on myoblasts, we next determined if DUX4-FL-induced γH2AX immunostaining also occurred in myotubes formed from primary human myogenic cells. We found that expression of DUX4-FL in myotubes, much as in myoblasts, was accompanied by punctate γH2AX immunostaining either when expressed exogenously via BacMam (Fig. 3) or from its endogenous promoter (Fig. 4).

Nuclei with increased γH2AX immunostaining were found in a large majority of myotubes with BacMam-mediated expression of DUX4-FL. In one experiment, we cultured control unaffected, primary human myogenic cells for 7 days in differentiation medium, added BacMam-DUX4-FL-V5 for an additional 2 days, and then carried out double immunostaining for DUX4-FL and γH2AX. In these cultures, 88.5% (46 out of 53) of the DUX4-FL-positive myotubes we examined contained myonuclei with γH2AX immunostaining, examples of which are shown in Fig. 3A–C. Not every DUX4-FL-positive myotube nucleus showed punctate γH2AX staining, though almost all showed immunostaining that was more intense than nearby DUX4-negative myotube nuclei (e.g. Fig. 3C). There did not appear to be a simple relationship between DUX4-FL and γH2AX staining patterns, because punctate γH2AX immunostaining was found in nuclei with both low and high DUX4-FL staining intensities within the same myotube (e.g. Fig. 3A,B). In contrast to the DUX4-FL-positive myotubes, a large majority of DUX4-FL-negative myotubes did not show obvious γH2AX immunostaining (Fig. 3C–E) as only 18.7% (14 out of 75) of the DUX4-negative myotubes we examined contained one or more myonuclei with γH2AX immunostaining, a distribution that differed from the DUX4-FL-positive group at *P*<0.0001 (Fisher's exact test).

Because previous studies had not examined if γH2AX accumulated upon endogenous DUX4 expression from its natural promoter, we next carried out double immunostaining for DUX4-FL and γH2AX in myotubes formed from human FSHD1 myogenic cells. Similar to the results with BacMam-mediated expression, we found that a majority of myotubes that expressed DUX4-FL from its endogenous promoter also showed γH2AX immunostaining (Fig. 4). For these studies, we examined cultures of primary human myogenic cells, termed 17Abic, that were obtained from a patient with FSHD1 (Homma et al., 2012). In previous studies, we showed that endogenous DUX4-FL protein was expressed in a small percentage (∼0.4%) of the nuclei in 17Abic cultures (Jones et al., 2012) and that DUX4-FL expression was almost exclusively in differentiated myocytes identified by sarcomeric myosin heavy chain expression (Himeda et al., 2014).

After 7 days in differentiation medium, we double immunostained 17Abic FSHD1 myotubes for endogenous DUX4-FL and γH2AX. We found one or more myotube nuclei with intense or multiply punctate γH2AX immunostaining in 72.3% (81 out of 122) of the endogenous DUX4-FL-positive myotubes that we examined, examples of which are shown in Fig. 4. In most myotubes, all or almost all nuclei that expressed endogenous DUX4-FL also showed intense or multiply punctate γH2AX staining (Fig. 4A,C,D). Again, however, there did not appear to be a simple relationship between endogenous DUX4-FL and γH2AX staining as, in some cases, obvious, often multiply punctate, γH2AX immunostaining was found in just one of the DUX4-positive nuclei within a myotube (e.g. nucleus indicated by arrow in Fig. 4B). In contrast to the endogenous DUX4-FL-positive myotubes, only a minority, 25% (12 out of 48), of the DUX4-negative myotubes we examined contained one or more myonuclei with γH2AX immunostaining. This distribution differed from the endogenous DUX4-FL-positive group at *P*<0.0001 (Fisher's exact test) but did not differ from the distribution found in our analysis of the DUX4-FL-negative myotubes noted above in the BacMam DUX4 study (*P*=0.49, Fisher's exact test).

### DUX4 and the Bax/Bak pathway

We next examined the role of the Bax/Bak pathway in DUX4-FL-induced processes. Altered nucleocytoplasmic transport and nuclear accumulation of γH2AX can be indicators of incipient cell death (Moeglin et al., 2019; Woerner et al., 2016) and DUX4-FL expression is known to induce caspase activation and cell death (Rickard et al., 2015). In many cellular contexts, the Bax/Bak pathway is known to mediate caspase activation as a step in the cell death pathway (Ke et al., 2018; Luo et al., 2020; Wei et al., 2001). Although Wallace et al. (2011) reported that DUX4-FL-induced caspase activation was partially inhibited by treatment with a Bax channel blocker, no further studies had directly examined the Bax/Bak pathway in DUX4-FL-induced pathogenesis.

**Fig. 5.**
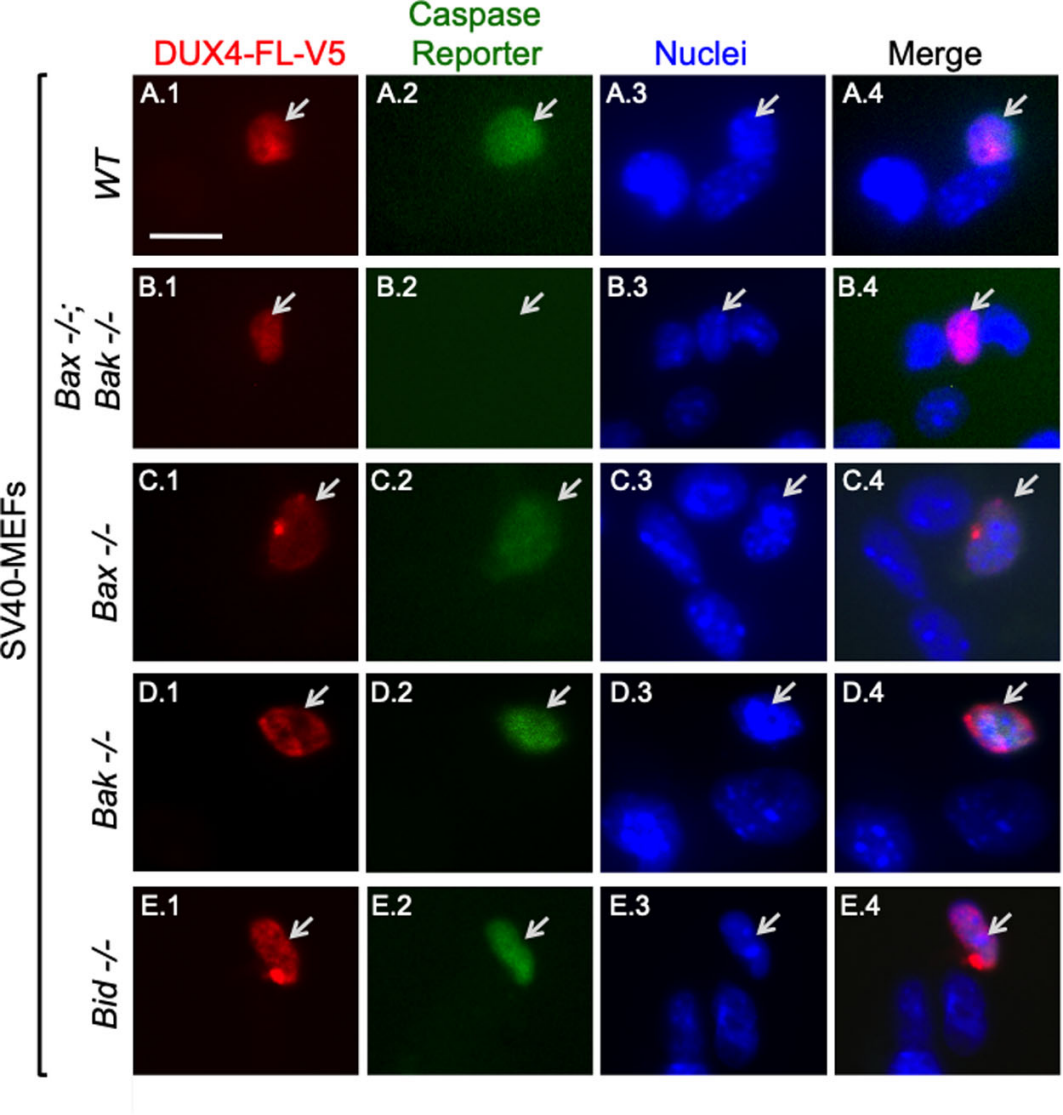
**Double knockout of *Bax* and *Bak* prevented DUX4-FL-induced caspase activation in SV40-immortalized MEFs.** After 48 h of incubation with BacMam-DUX4-FL-V5, cultures of wild-type (row A, *WT*), double knockout *Bax-/-;Bak-/-* (row B), and single knockout *Bax-/-* (row C), *Bak-/-* (row D), or *Bid-/-* (row E) SV40-MEFs were incubated for 1 h with NucView488 (Caspase Reporter, green) and subsequently immunostained for DUX4-FL-V5 (mAb E5-5, red). In cells with active caspase 3/7 (DEVDase), the reporter is designed to develop green fluorescence upon caspase cleavage and then accumulate in nuclei. The images are representative of the salient finding for each genotype, i.e. that DUX4-FL expression was accompanied by caspase activation in a subset (10–20%) of MEFs with single knockouts, whereas double knockout of *Bax* and *Bak* almost completely eliminated caspase activation. Arrows indicate cells that expressed DUX4-FL-V5. See Table 1 for quantitative analysis. Scale bar in panel A.1: 15 µM.

For these studies, we used SV40-immortalized mouse embryonic fibroblasts (MEFs) (Wei et al., 2001). The MEF cell lines included wild type, double knockout (DKO) for *Bax* and *Bak*, and single knockouts for *Bax*, *Bak*, and *Bid.* Each line was grown independently, treated with BacMam-DUX4-FL-V5 for 48 h, incubated with the NucView488 caspase reporter for 1 h, and finally immunostained for DUX4-FL-V5 (Fig. 5). [The NucView488 reagent is non-fluorescent until cleaved by caspase 3/7 (DEVDase), at which time it develops green fluorescence and accumulates in nuclei.]

**Table 1.**
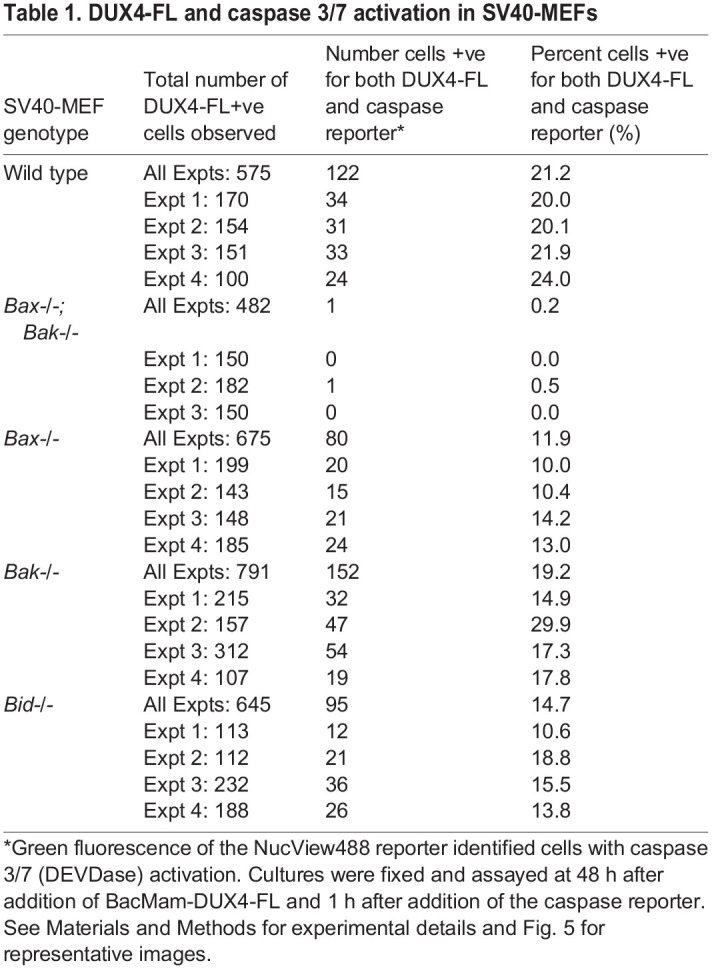
DUX4-FL and caspase 3/7 activation in SV40-MEFs

As shown by representative images in Fig. 5 and quantified in Table 1, DUX4-FL-V5 expression was accompanied by caspase activation in ∼10–20% of the cells of all tested genotypes, with the notable exception of *Bax-/-;Bak-/-* double knockout cells where we found only one cell (out of 482 examined) that was positive for both caspase reporter and DUX4-FL-V5. We found that ∼20% of wild-type and single knockout *Bak-/-* cells were double-positive for caspase and DUX4-FL-V5, whereas single knockout *Bid-/-* cells, at ∼15%, and single knockout *Bax-/-* cells, at ∼10%, consistently showed lower percentages of cells that were double-positive for caspase and DUX4-FL-V5 (Table 1). As expected (Homma et al., 2015; Mitsuhashi et al., 2018), caspase activation was negligible upon 48 h expression of non-toxic DUX4-S-V5. We found NucView488 fluorescence in only four out of 334 (1.2%) DUX4-S-positive wild-type MEFs.

Because *Bax-/-;Bak-/-* double knockout SV40-MEFs were almost entirely resistant to DUX4-FL-induced caspase activation, we next asked whether the DKO MEFs were similarly resistant to DUX4-FL-induced alteration of nucleocytoplasmic transport or nuclear accumulation of γH2AX. To examine nucleocytoplasmic transport, the shuttle-GFP plasmid was transfected into wild-type and DKO MEFs and 24 h later BacMam-DUX4-FL-V5 was added for an additional 48 h. However, there were two characteristics of the MEFs that rendered the shuttle-GFP assay impracticable. First, the MEFs had small cytoplasmic volumes making it difficult to confidently measure cytoplasmic fluorescence intensity for comparison to nuclear intensity (supplemental data, Fig. S2). Second, unlike in primary human myoblasts (Fig. 1A), the shuttle-GFP reporter accumulated to high levels in all wild-type and DKO SV40-MEF nuclei whether or not DUX4-FL was expressed (Fig. S2). Therefore, though nuclear shuttle-GFP fluorescence did not appear to be decreased by DUX4-FL, the unfavorable morphology and high nuclear accumulation of the reporter in the absence of DUX4 made it impossible to determine if DUX4-FL expression led to additional accumulation of shuttle-GFP beyond the high level already found in DUX4-negative MEFs.

**Fig. 6.**
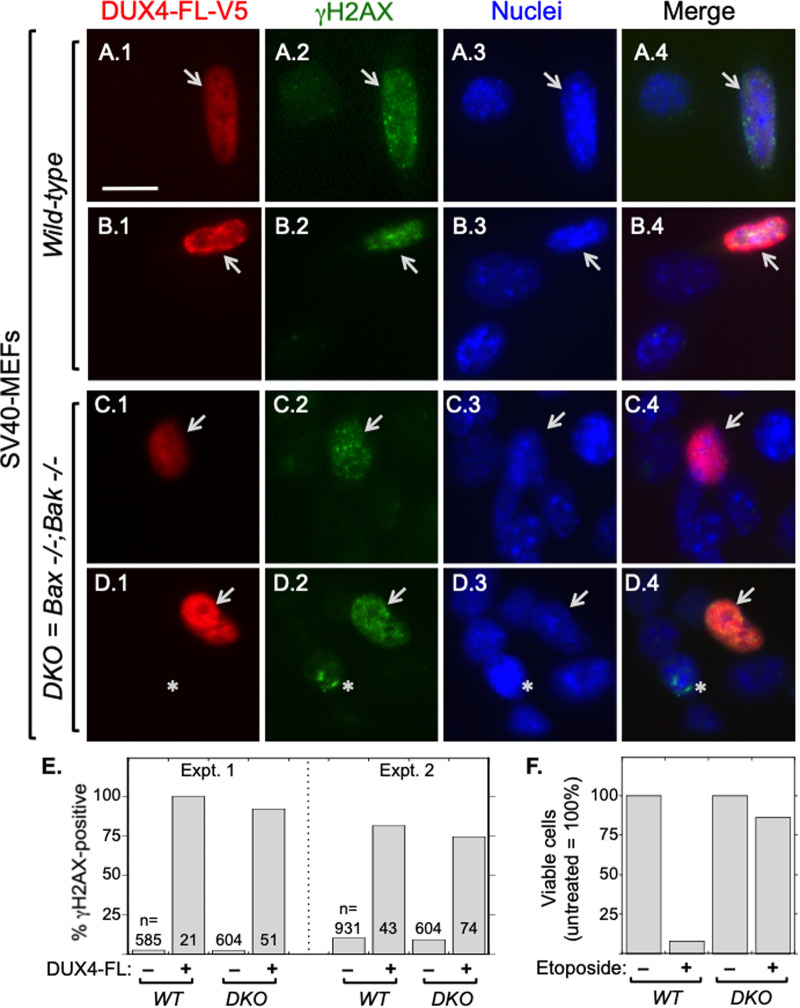
**Expression of DUX4-FL was accompanied by γH2AX immunostaining in both wild-type and double knockout *Bax-/-;Bak-/-* SV40-immortalized MEFs.** After 48 h of incubation with BacMam-DUX4-FL-V5, cultures of *wild-type* (rows A, B) and double knockout *Bax-/-;Bak-/-* (rows C, D) SV40-immortalized MEFs were double immunostained for DUX4-FL (red, rabbit mAb E5-5) and γH2AX (green, mouse mAb 3F2). Arrows indicate cells that expressed DUX4-FL-V5; Asterisk in row D indicates one of the minority of DUX4-FL-negative cells with a positive γH2AX signal. Scale bar in panel A.1: 15 µM. (E) Quantitation. In two independent experiments, >75% of DUX4-FL-positive nuclei of both wild-type (*WT*) and double knockout *Bax-/-;Bak-/-* (*DKO*) SV40-immortalized MEFs showed γH2AX immunostaining, whereas <10% of the DUX4-FL-negative *WT* and *DKO* nuclei showed γH2AX immunostaining. (F) Treatment with 100 µM etoposide for 18 h killed almost all wild-type (*WT*) MEFs, but had little effect on the viability of double knockout *Bax-/-;Bak-/-* (*DKO*) MEFs, thereby confirming the expected difference in etoposide sensitivity for cells of the two genotypes.

Finally, we found that increased γH2AX immunostaining upon DUX4-FL expression occurred in DKO, as well as in wild-type, MEFs (Fig. 6A–D). Quantitation of two independent experiments (Fig. 6E) showed that >75% of both wild-type and DKO MEFs showed increased γH2AX immunostaining after 48 h of BacMam-mediated DUX4-FL expression. In contrast, <10% of the DUX4-FL-negative wild-type and DKO MEFs showed the more intense or multiply punctate γH2AX immunostaining pattern, one example of which is shown in Fig. 6D.2. We also verified cell genotypes by showing that, as expected (Wei et al., 2001; Ke et al., 2018), treatment with etoposide, an inhibitor of DNA topoisomerase II, efficiently killed wild-type, but not DKO, SV40-MEFs (Fig. 6F). We conclude that accumulation of γH2AX in DKO cells following DUX4-FL expression occurs independently of caspase activation and the Bax/Bak pathway.

## DISCUSSION

In this work, we (i) examined how aberrant DUX4 expression affected nucleocytoplasmic protein transport, (ii) extended previous studies (DeSimone et al., 2019; Dmitriev et al., 2016; Shadle et al., 2019) of DUX4-induced DNA damage as identified by nuclear accumulation of γH2AX, and (iii) determined that the Bax/Bak pathway mediated DUX4-induced caspase activation.

For nucleocytoplasmic transport, we found that the percentage of myoblasts with nuclear accumulation of the shuttle-GFP reporter (Woerner et al., 2016) was increased by exogenous DUX4-FL expression, consistent with an average decrease in nuclear protein export. Because wide variations in nuclear-to-cytoplasmic shuttle-GFP fluorescence were found among populations of both DUX4-FL-positive and -negative myoblasts, it was not possible to identify a particular cell as DUX4-FL-positive or -negative simply from its shuttle-GFP distribution. The extent to which nucleocytoplasmic transport (i.e. shuttle-GFP fluorescence) responds to DUX4-FL might depend on the level and/or duration of DUX4-FL expression within a cell, but further work is needed to test at different durations of DUX4-FL expression and in myotubes as well as in myoblasts.

In neurons, disrupted nucleocytoplasmic transport and altered localization of nuclear envelope proteins is associated with nuclear aggregation or clearance of TDP-43, as observed, e.g. in amyotrophic lateral sclerosis (Chou et al., 2018; Gasset-Rosa et al., 2019; Moore et al., 2020). In myogenic cells, we have shown that, upon DUX4-FL expression, a percentage of myoblast and myotube nuclei show nuclear aggregation or clearance of TDP-43 (Homma et al., 2015; 2016); and our current study suggests that DUX4-FL expression may, at least partially, disrupt nucleocytoplasmic transport in myogenic cells. Of note, plasmid-mediated overexpression of DUX4 in human epithelial carcinoma HEp-2 cells leads to mis-localization of the inner nuclear envelope protein, emerin (Kowaljow et al., 2007). Whether DUX4-FL expression alters localization of emerin and other nuclear envelope proteins in myogenic cell nuclei remains to be determined.

Dmitriev et al. (2016) were the first to show, as part of their extensive analysis of DUX4-induced DNA damage, that plasmid-mediated expression of DUX4 was accompanied by increased γH2AX in human primary myoblast nuclei. We confirmed that γH2AX immunostaining was increased in human primary myoblasts upon exogenous expression of DUX4, in our case via BacMam. We also extended previous studies (DeSimone et al., 2019; Dmitriev et al., 2016; Shadle et al., 2019) by analyzing DUX4-induced accumulation of γH2AX in myotube nuclei and in Bax/Bak double-knockout SV40-MEFs.

In the subset of nuclei with punctate DUX4-FL immunostaining, the γH2AX and DUX4-FL puncta did not overlap, indicating that DUX4 did not localize to sites of DNA damage. In previous work with FSHD1 myotubes, we showed that DUX4 induces nuclear co-aggregates of TDP-43 and FUS that do not include DUX4 (Homma et al., 2015; 2016). Because TDP-43 and FUS co-localize in FSHD1 myonuclei that express endogenous DUX4 (Homma et al., 2016), we would predict that TDP-43, like FUS (DeSimone et al., 2019), should co-localize with γH2AX foci, which would be consistent with the role of TDP-43 in the repair of DNA double-strand breaks in neurons (Mitra et al., 2019).

As in myoblasts, expression of DUX4-FL in post-mitotic myotubes, either from its endogenous promoter or BacMam-mediated, generated increased histone γH2AX immunostaining. The staining patterns of DUX4 and γH2AX often varied among the nuclei within a single myotube, indicating that progression of DUX4-initiated events occurs at different rates among different myonuclei. Such nucleus-to-nucleus variation might be expected given that endogenous DUX4 expression can begin in a single myotube nucleus (Rickard et al., 2015).

DUX4 expression is one of several mechanisms that lead to increased γH2AX in skeletal muscle cells. For example, myogenic cells of the mouse C_2_C_12_ immortalized line show a transient increase in γH2AX immunostaining at the onset of myotube formation that returns to baseline within 48 h (Larsen et al., 2010). All of our studies of human myotubes and DUX4 expression took place at least 7 days after initiation of differentiation when γH2AX staining was infrequent in DUX4-FL-negative myotubes. Increased γH2AX immunostaining also occurs in mouse myotubes treated with H_2_O_2_ to generate oxygen stress (Narciso et al., 2007) or with the DNA alkylating agent methyl methanesulfonate to generate DNA breaks (Fortini et al., 2012). In humans, increased γH2AX immunostaining in myofibers has been associated with sporadic inclusion body myositis (Nishii et al., 2011) and obesity (Dungan et al., 2020).

In most cellular contexts, histone γH2AX immunostaining is a reliable biomarker for double strand DNA breaks (D'Abrantes et al., 2018; Georgoulis et al., 2017; Moeglin et al., 2019; 2021) and, based on the work of Dmitriev et al. (2016), the DUX4-FL-induced increase of histone γH2AX in myoblasts is indeed a marker for genotoxicity characterized by double strand DNA breaks. In proliferating mouse embryonic stem cells, however, γH2AX has been associated with global chromosome decondensation (Banáth et al., 2009; Turinetto and Giachino, 2015), and DUX4 expression also has been linked to global chromosomal remodeling in early stage embryos and upon ectopic expression in myogenic cells (Choi et al., 2016; Hendrickson et al., 2017; Liu et al., 2019; Resnick et al., 2019). Whether such chromosomal changes may also contribute in some degree to the γH2AX increase in myoblasts or myotubes remains to be determined. It would also be informative to determine if DUX4-FL-induced γH2AX immunostaining is reversible upon cessation of DUX4-FL expression, as well as to determine the frequency of γH2AX-positive nuclei in FSHD patient myofibers in comparison to the low frequency of TUNEL-positive myonuclei (Hauerslev et al., 2013; Sandri et al., 2001; Statland et al., 2015).

DUX4-FL-induced activation of caspase 3/7 (DEVDase) was blocked in SV40-immortalized MEFs by double knockout of *Bax* and *Bak*, but not by single knockouts of *Bax*, *Bak*, or *Bid*. Because *Bid-/-* and *Bax-/-* MEFs had lower percentages of cells that were double-positive for caspase and DUX4-FL-V5 than *wild-type* or *Bak-/-* MEFs, it is likely that DUX4-FL-induced caspase activation is partially inhibited by deletion of *Bid* or *Bax*. This possibility is consistent with a report that DUX4-FL-induced caspase activation is partially inhibited by a Bax channel inhibitor (Wallace et al., 2011). Further work will be needed to identify mechanisms that link DUX4 expression to engagement of the pro-death Bax/Bak pathway and subsequent caspase activation.

DUX4-FL-induced genotoxicity marked by γH2AX accumulation occurred upstream and independent of Bax/Bak and caspase activation in DKO MEFs. The work of Dmitriev et al. (2016) shows that γH2AX accumulation can be driven by a DUX4-initiated increase in reactive oxygen species, which is consistent with the caspase-independent mechanism. DUX4-FL-induced nuclear aggregation of TDP-43 also appears to be independent of caspase activation (Homma et al., 2015), but work is needed to determine if other DUX4-induced changes require the Bax/Bak pathway and/or caspase activation. In SV40-MEFs, interactions of SV40 large T and small t antigens with p53, pRb-E2F, and phosphatase PP2A (Bocchetta et al., 2008; Cantalupo et al., 2009) could affect DUX4-induced events, so studies are needed to confirm that Bax and Bak are required for DUX4-induced caspase activation in primary human myogenic cells.

As potential therapies for FSHD, multiple groups are developing technologies to inhibit the function or expression of DUX4-FL (e.g. Bosnakovski et al., 2019; Bouwman et al., 2021; Das and Chadwick, 2021; Himeda et al., 2020; Lim et al., 2020, 2021; Lu-Nguyen et al., 2021; Mariot et al., 2020; Mellion et al., 2021; Oliva et al., 2019; Rashnonejad et al., 2020; Šikrová et al., 2021). Direct targeting of DUX4 will likely be the primary therapeutic strategy for FSHD, because once begun, any technique that inhibits DUX4 expression or function should block multiple pathogenic changes that are dependent on DUX4 transcriptional activity (Mitsuhashi et al., 2018). However, DUX4 expression in FSHD appears to occur in bursts within an individual nucleus, and this episodic expression appears to initiate changes that persist after DUX4 expression ends (Rickard et al., 2015). If such persistent changes are of significance to disease progression, or if the primary therapeutic strategy produces less-than-complete inhibition of DUX4, then secondary strategies aimed at downstream targets, e.g. genotoxicity, the Bax/Bak pathway, or caspase activation, could prove of benefit when combined with direct targeting of DUX4.

### Conclusions

Our results suggest that ectopic DUX4-FL expression can alter nucleocytoplasmic protein transport and show that endogenous DUX4 expression in FSHD1 myotubes induces nuclear accumulation of γH2AX, a marker for DNA damage. Furthermore, we found that double knockout of Bax and Bak blocked DUX4-FL-induced activation of caspase 3/7 (DEVDase), but not DUX4-FL-induced accumulation of γH2AX. These studies suggest possibilities for further investigation of DUX4-induced pathogenic mechanisms.

## MATERIALS AND METHODS

### Cells and culture

Previous publications have described institutional approvals, isolation, culture conditions, and authentication protocols for the human primary myogenic cells from an unaffected donor (09Ubic) and an FSHD1 donor (17Abic) that were used here (Himeda et al., 2014; Homma et al., 2012; 2015; 2016; Jones et al., 2012). Growth medium for replicating myoblasts consisted of Ham's F-10 medium (cat. 11550043, Thermo Fisher Scientific) supplemented with 18% fetal bovine serum (cat. 16000044, Gibco Thermo Fisher Scientific), 1.2 mM CaCl_2_ (cat. J62905.AP, Thermo Fisher Scientific), 10 ng/ml heat stable, recombinant human bFGF (cat. PHG0369, Thermo Fisher Scientific), 1% antibiotics/antimycotics (cat. 15240062, Thermo Fisher Scientific), and 1 µM dexamethasone (cat. A17590.03, Thermo Fisher Scientific). Differentiation medium to induce myotube formation consisted of a 50:50 mixture of (i) complete Skeletal Muscle Differentiation Medium (cat. C23061, PromoCell, Heidelberg, Germany) and (ii) DMEM (cat. 11965092, Thermo Fisher Scientific) supplemented with 2% horse serum (cat. 16050122, Thermo Fisher Scientific), 2 mM glutamax (cat. 35050061, Thermo Fisher Scientific), 10 mM HEPES (cat. 15630080, Thermo Fisher Scientific), and 1 mM sodium pyruvate (cat. 11360070, Thermo Fisher Scientific).

All human primary myogenic cells were >90% desmin-positive and were used at less than 45 population doublings. As we noted previously (Homma et al., 2016), all human “cells were anonymized prior to receipt with no personal identifying information available to us. In addition, the cells had been produced prior to our study from muscle biopsies collected under protocols approved by the appropriate institution that included informed donor consent and approval to publish results in accordance with standards of the Helsinki Declaration. Because our studies were of human cells that were obtained from a cell bank and for which personal identification data were not obtainable by us, the studies were classified as exempt from Human Studies review by the Boston University Institutional Review Board in accordance with the US Department of Heath and Human Services policy”.

SV40-immortalized MEFs were obtained from the American Type Culture Collection, including *wild-type* (ATCC CRL-2907), *Bax-/-* (ATCC CRL-2910), *Bak-/-* (ATCC CRL-2912), double knockout *Bax-/-;Bak-/-* (ATCC CRL-2913), and *Bid-/-* (ATCC CRL-2911) cells. The MEF lines were grown in Ham's F-10 medium (Gibco) supplemented with 10% fetal bovine serum (Gibco). Authentication included ATCC certification, immunoblotting (Nowak et al., 2004), and testing to confirm that double knockout *Bax-/-;Bak-/-* MEFs, but not single knockouts or wild type, were resistant to killing by etoposide (Fig. 6F) (Ke et al., 2018; Wei et al., 2001). For immunocytochemistry, all cells were grown on porcine gelatin-coated four-well or eight-well permanox chamber slides (cat. 177437 and 177445, Thermo Fisher Scientific, Waltham MA, USA) or 35 mm diameter cell culture dishes.

### Antibodies

DUX4-FL was detected with rabbit anti-DUX4-FL mAb E5-5 (cat. ab124699, Abcam, Cambridge, MA, USA), which reacts with a C-terminal domain epitope specific to DUX4-FL (Geng et al., 2011) and was used at 1:200 dilution. The V5 epitope tag on BacMam-expressed DUX4-FL and DUX4-S was detected using either a mouse mAb (cat. R960-25, Thermo Fisher Scientific) used at 1:500 or a rabbit pAb (cat. AB3792, EMD Millipore, Burlington, MA, USA) used at 1:300. Phospho(Ser139)-H2AX (γH2AX) was assayed with three different antibodies (which gave similar results) as specified in the text: (i) mouse mAb 3F2 (cat. MA1-2022, Thermo Fisher Scientific); (ii) mouse mAb JBW301 (cat. 05-636, Sigma-Aldrich); and (iii) rabbit mAb 20E3 (cat. 9718, Cell Signaling Technology, Danvers, MA, USA), each of which was used at 1:200. Desmin was detected with mouse mAb DE-U-10 (cat. D1033, Sigma-Aldrich) used at 1:500.

Each primary antibody was validated based on one or more methods, including prior use in multiple published studies with the same mAb or lot of polyclonal antiserum, manufacturer's validation assays including knockouts, generation of expected immunofluorescence staining patterns on wild-type and knockout cells, detection of appropriate band size on immunoblots without detection of non-specific bands, and detection of recombinant protein when expressed in cells that normally do not express the protein. Primary antibody binding was visualized with appropriate species-specific secondary antibodies (Life Technologies) conjugated to either Alexa Fluor 488 or Alexa Fluor 594 and used at 1:500. Nuclei were stained with bisbenzimide (cat. 14530, Sigma-Aldrich). Slides were mounted in Vectashield (cat. H-1200-10, Vector Laboratories, Burlingame, CA, USA).

### BacMam virus vectors

DUX4-FL and DUX4-S, both tagged with C-terminal V5 epitopes, were expressed under control of the human CMV-IE1 promoter in BacMam viruses generated as described (Fornwald et al., 2016; Homma et al., 2015; 2016; Mansouri et al., 2016; Mitsuhashi et al., 2019). Viral supernatants were used without further purification. DUX4-FL or -S became detectable by immunostaining at 24–48 h after addition of the virus, and, in different experiments, the amount of BacMam-containing supernatant added to host cells was adjusted so that the percentage of DUX4-positive host cells that were detected by immunostaining ranged from 1–30% at 48 h after addition.

### Immunostaining

Cultures were washed twice with PBS and fixed with 2% paraformaldehyde in PBS for 10 min at room temperature. Fixed cultures were washed three times over 10 min; additionally permeabilized with 0.5% Triton X-100 for 15 min at room temperature; and incubated for 60 min at room temperature in blocking solution containing 2% horse serum, 2% normal goat serum (Thermo Fisher Scientific), 2% bovine serum albumin, and 0.1% Triton X-100. For double immunostaining, fixed and blocked cultures were incubated overnight at 4°C with two primary antibodies of different species (e.g. mouse anti-γH2AX and rabbit anti-DUX4-FL) diluted as noted in blocking solution. The following day, cells were rinsed three times with PBS and incubated for 1 h at room temperature with the appropriate secondary antibodies (e.g. Alexa Fluor 488-labeled anti-mouse and Alexa Fluor 594-labeled anti-rabbit) diluted 1:500 in blocking solution. For double immunostaining, the cultures were subsequently incubated for 2 h at room temperature with the second primary antibody, washed as above, and incubated with the second secondary antibody as above. All solutions were made with phosphate-buffered saline (PBS), except when using anti-γH2AX antibodies in which case all solutions used subsequent to paraformaldehyde fixation were made with Tris-buffered saline (TBS) unless stated otherwise.

### Shuttle-GFP plasmid and transfection

The plasmid for expression of shuttle-GFP (Woerner et al., 2016), a reporter for nucleocytoplasmic transport, was a gift of Ulrich Hartl and Nadine Wischnewski (Max Planck Institute of Biochemistry, Martinsreid, Germany). The shuttle-GFP plasmid was transfected into human primary myogenic cells or SV40-immortalized MEFs using X-treme GENE 9 HP (cat. XTGHP-RO, Roche Diagnostics, Indianapolis, USA), Turbofect (cat. R0533, Thermo Fisher Scientific), or TransIT-LT1 (cat. MIR2304, Mirus Bio, Madison, WI, USA) following the manufacturer's instructions. At 24 h after transfection with the shuttle-GFP plasmid, the transfected cells were exposed to BacMam-DUX4 viruses for an additional 48 h, at which time the cells were fixed and immunostained for the V5 epitope tag as above. As a positive control for shuttle-GFP function, we confirmed that GFP accumulated in the nucleus when cultures were treated with a known inhibitor of nuclear export, Leptomycin B (cat. 9676, Cell Signaling Technology), used at 10 nM for 3 h at 37°C.

### Quantitative microscopy

Two methods were used to quantify shuttle-GFP experiments. In the first method, observers were provided images of shuttle-GFP-expressing cells and asked to score each cell on a five-point scale as follows: (1) Cytoplasm much more intense than nucleus. (2) Cytoplasm distinct from and somewhat more intense than nucleus. (3) Nucleus and cytoplasm have similar GFP fluorescence intensity. (4) Nucleus distinct from and somewhat more intense than cytoplasm. (5) Nucleus much more intense than cytoplasm. Images were presented in a randomized order and observers did not know if a cell was DUX4-negative or DUX4-positive or if it had been treated with leptomycin B. Multiple observers showed high agreement on scores. In one group of 141 cells, for example, four observers agreed within one point on 95.0% (*n*=134) of the cells with complete agreement on 49.6% (*n*=70) of cells. For three cells, scores differed by two, and for four cells (2.8%), one or more observers was unable to classify the cell. In the second method to quantify shuttle-GFP fluorescence, the Plot Profile function of NIH ImageJ software (v. 1.51.s) was used to determine average fluorescence intensities in the nucleus and in adjoining cytoplasm, from which the nuclear-to-cytoplasmic fluorescence ratio was then calculated for each cell.

To determine cell viability in etoposide studies, the %Area function of NIH ImageJ was used to analyze the extent of bisbenzimide-stained nuclei in 20X images of treated versus untreated cultures. Images of immunostained cultures were acquired using a Nikon E800 microscope with a Spot camera and software version 5.1 (Diagnostic Instruments Inc., Sterling Heights, MI, USA). Data in Fig. 1 were obtained from cells in four independent shuttle-GFP/DUX4-FL co-expression experiments. Experiments illustrated in Figs 2–6, Figs S1 and S2 were independently replicated two or more times. Statistical analyses were carried out with either Kaleidagraph (v. 4.1.3, Synergy Software, Reading, PA, USA) or GraphPad Prism 7 (GraphPad Software, LaJolla, CA, USA). Appropriate two-tailed *t*-tests, ANOVA, or chi-square comparisons were used as noted in figure legends and text. Equal variance was not assumed.

### Caspase activity reporter assay

To identify cells with active caspase 3/7 (DEVDase), we used the NucView488 reagent (cat. 30029, Biotium, Fremont, CA, USA), which is non-fluorescent until cleaved by caspase 3/7, at which time it produces green fluorescence and accumulates in nuclei. After 48 h of incubation with BacMam-DUX4, unfixed cultures were incubated with 5 µM NucView488 in growth medium for 1 h at room temperature in the dark per the manufacturer's instructions. Cultures were then fixed with paraformaldehyde, permeabilized, and immunostained for DUX4 as described above.

## Supplementary Material

Supplementary information
