# Supplementary material for: Downstream events initiated by expression of FSHD-associated DUX4: Studies of nucleocytoplasmic transport, γH2AX accumulation, and Bax/Bak-dependence
Source: Biol Open. 2022 Feb 22;11(2):bio059145. doi: 10.1242/bio.059145 (PMC8890089; doi:10.1242/bio.059145)
Supplement: Supplementary information [file biolopen-11-059145-s1.pdf]

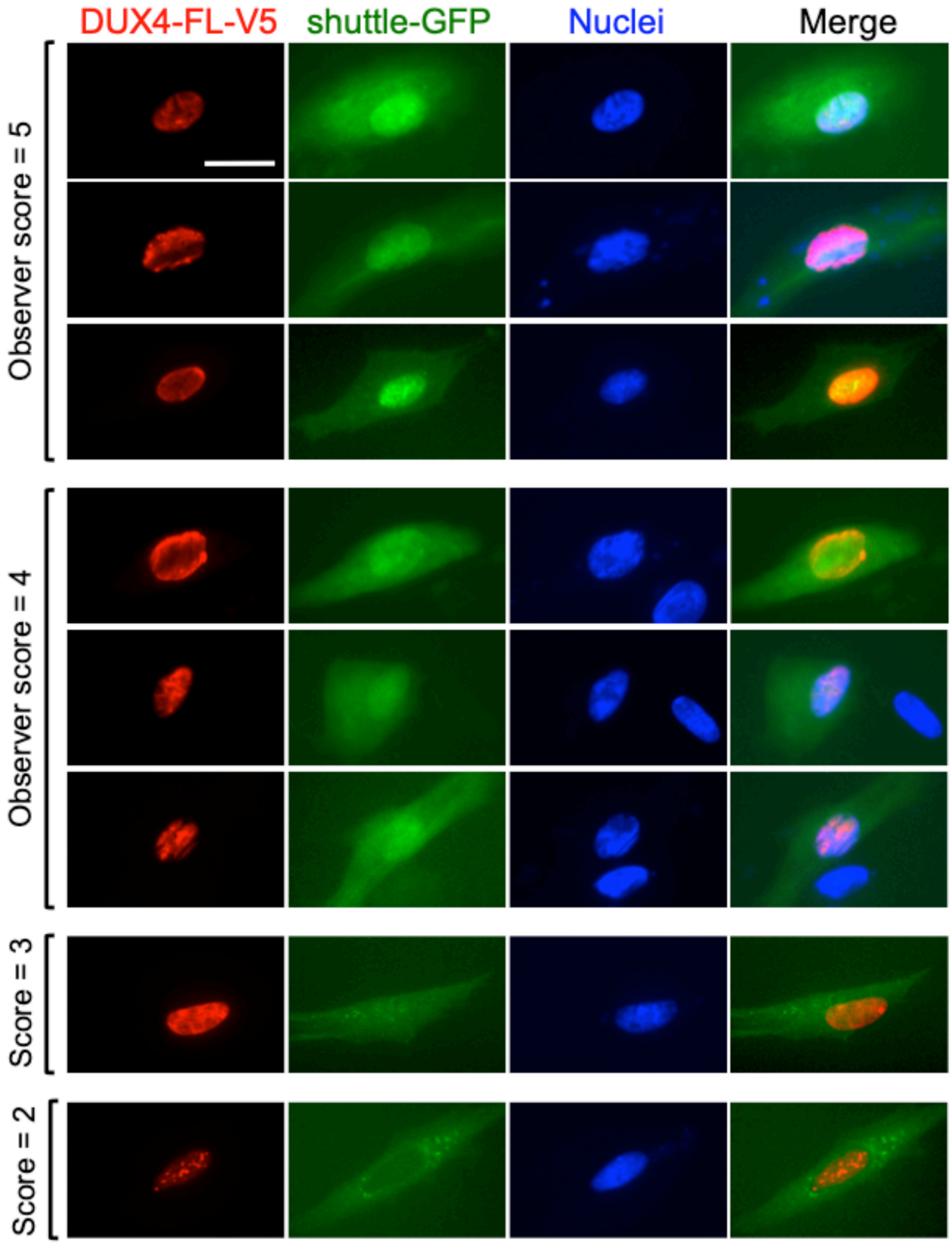
DUX4-FL-V5
shuttle-GFP
Nuclei
Merge
Observer score = 5
Observer score = 4
Score = 3
Score = 2

Fig. S1. Examples of shuttle-GFP reporter distribution in human primary myoblasts that also had BacMam-mediated DUX4-FL-V5 expression. Higher shuttle-GFP fluorescence intensity in the nucleus than in the cytoplasm was the most frequently observed pattern (i.e., observer scores 4 or 5). Less frequently observed patterns included approximately equal nuclear and cytoplasmic fluorescence (observer score = 3) and higher cytoplasmic than nuclear fluorescence (observer score = 2). In the ma text, see Materials and Methods for the observer scoring system and Fig. 1 for quantitation of all results. Bar=15 μM.

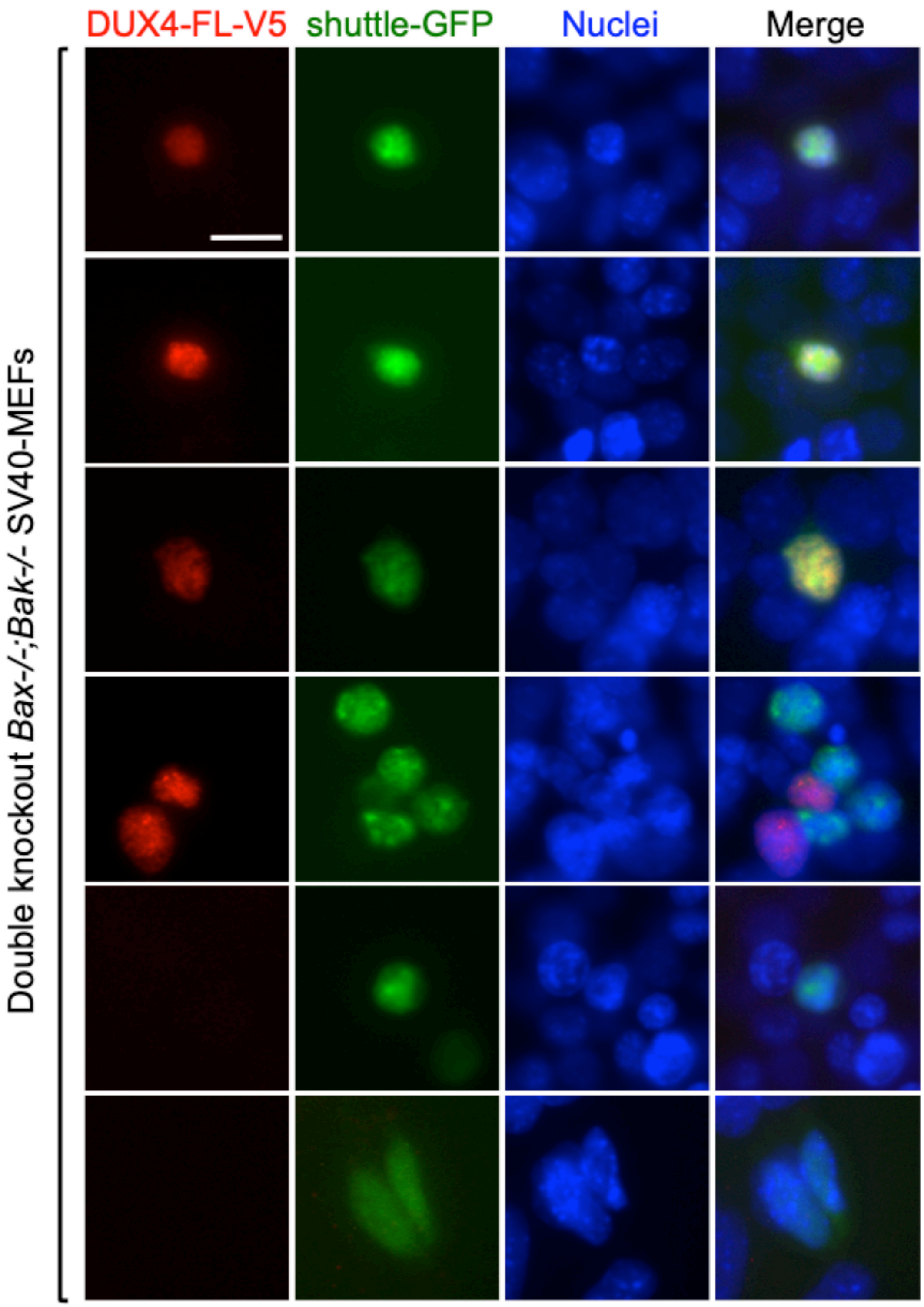
DUX4-FL-V5
shuttle-GFP
Nuclei
Merge
Double knockout Bax-/-;Bak-/- SV40-MEFs

Fig. S2. Examples of shuttle-GFP reporter distribution in *Bax-/-;Bak-/-* (double knockout) SV40-immortalized mouse embryonic fibroblasts (MEFs) in the presence and absence of BacMam-mediated DUX4-FL-V5 expression. As noted in the text, MEFs had little cytoplasm and shuttle-GFP fluorescence was high in all nuclei with or without DUX4-FL expression. Similar results were seen with wild-type MEFs. Shuttle-GFP fluorescence in nuclei did not appear to be decreased by DUX4-FL expression, but, as discussed in the text, the unfavorable cell morphology and high nuclear accumulation of the reporter in the absence of DUX4 made it impossible to determine if DUX4-FL expression led to additional accumulation of shuttle-GFP beyond the high level already found in DUX4-negative MEFs. Bar=15 μM.
